# Relationship Between Gut Bacteria and Levodopa Metabolism

**DOI:** 10.2174/1570159X21666221019115716

**Published:** 2023-05-18

**Authors:** Kaifei Xu, Shuo Sheng, Feng Zhang

**Affiliations:** 1 Key Laboratory of Basic Pharmacology of Ministry of Education and Joint International Research Laboratory of Ethnomedicine of Ministry of Education and Key Laboratory of Basic Pharmacology of Guizhou Province and Laboratory Animal Center, Zunyi Medical University, Zunyi, Guizhou, China;; 2 The Collaborative Innovation Center of Tissue Damage Repair and Regeneration Medicine of Zunyi Medical University, Zunyi, Guizhou, China

**Keywords:** Parkinson's disease, levodopa metabolism, gut bacteria, therapeutic efficacy, *Enterococcus faecalis*, *Eggerthella lenta*, *Clostridium sporogenes*

## Abstract

Parkinson's disease (PD) is one of the most common neurodegenerative diseases, characterized by the reduction of dopamine neurons in the substantia nigra. Levodopa, as a dopamine supplement, is the gold-standard therapeutic drug for PD. The metabolism of levodopa in the periphery not only decreases its bioavailability but also affects its efficacy. Thus, it is necessary to investigate how levodopa is metabolized. A growing number of studies have shown that intestinal bacteria, such as *Enterococcus faecalis*, *Eggerthella lenta* and *Clostridium sporogenes*, could metabolize levodopa in different ways. In addition, several pathways to reduce levodopa metabolism by gut microbiota were confirmed to improve levodopa efficacy. These pathways include aromatic amino acid decarboxylase (AADC) inhibitors, antibiotics, pH and (S)-α-fluoromethyltyrosine (AFMT). In this review, we have summarized the metabolic process of levodopa by intestinal bacteria and analyzed potential approaches to reduce the metabolism of levodopa by gut microbiota, thus improving the efficacy of levodopa.

## 1 INTRODUCTION

Parkinson's disease (PD) is one of the most common neurodegenerative diseases worldwide, mainly in middle-aged and elderly people older than 65 years of age [1]. The main pathological change in PD is the degenerative death of dopamine neurons in the midbrain substantia nigra, resulting in the corresponding decrease in striatal dopamine content. Although the exact etiology of this pathology remains unclear, genetic mutations, environmental factors, ageing and oxidative stress might be involved in dopamine neurodegeneration. Clinical manifestations include motor symptoms, such as quiescent tremor, bradykinesia, myotonia and postural gait disturbances [2], while patients could have non-motor symptoms, such as depression, constipation and sleep disturbances.

Levodopa is currently the gold-standard therapeutic agent for PD. As a dopamine precursor, levodopa can cross the blood-brain barrier (BBB) and enter the cytoplasm of dopamine neurons in the brain, where it is metabolized to dopamine by the action of aromatic amino acid decarboxylase (AADC). Although levodopa is effective in improving motor symptoms of PD patients, the response to levodopa varies with disease progression as the duration of treatment continues. Within 5-10 years of drug use, most patients experience the declined therapeutic efficacy of levodopa and various degrees of side effects of levodopa, such as levodopa-induced dyskinesia (LID) [1, 3]. The clinical manifestations of LID are choreiform movements, dystonia tardive dyskinesia and simple repetitive involuntary movements, which severely affect the quality of life of PD patients in the later stages [4]. Levodopa is mainly decarboxylated by AADC to dopamine in peripheral organs, such as the small intestine and liver, before levodopa enters the brain [5]. Therefore, levodopa is often used in combination with an AADC enzyme inhibitor, such as carbidopa, to minimize its peripheral metabolism. Given that levodopa is usually administered orally or enterally, it is suggested that gut microbiota might influence the therapeutic effect of levodopa [6, 7].

The intestinal tract plays an important role in people's lives, and the gastrointestinal tract of a healthy person hosts a large variety of microorganisms called the gut microbiota. A great number of bacteria are colonized in human intestines, about 40 trillion, 10 times the number of human cells, and the total number of genes is about 150 times the number of human genes [8]. Numerous studies have shown that the intestinal microbiota and its metabolites play a critical role in various health and disease states. Host immune system and brain development, metabolism, behavior, stress and pain response have been illuminated to be closely associated with microbiota disorders [9-13]. Additional studies have indicated that gut microbes could affect the efficacy of drugs [14, 15]. For example, it has been shown that oral or enteral administration of levodopa, even in combination with AADC inhibitors, still leaves 56% of levodopa in peripheral metabolism [16], decreasing drug bioavailability and causing side effects. The metabolism of levodopa by gut microbiota leads to reduced bioavailability and, therefore, a progressive increase in the dose of the drug used as the duration of treatment increases, leading to serious drug side effects. If one could reduce the metabolism of levodopa by intestinal bacteria, its bioavailability would be increased, likely leading to a reduction of side effects and an improvement in the patient’s quality of life.

## 2 METABOLISM OF LEVODOPA IN THE INTESTINAL TRACT

### 2.1 Metabolism of Levodopa by Peripheral Enzyme

During treatment with levodopa, it is transported into the brain by neutral amino acid transporter proteins across BBB. However, levodopa could be metabolized in the periphery by two pathways before it enters the brain. One is AADC and the other one is catechol-O-methyltransferase (COMT). AADC metabolizes levodopa into dopamine in the periphery, and dopamine does not enter the brain, thus affecting intestinal motility and causing intestinal damage. Therefore, levodopa is often used clinically in combination with AADC inhibitors, such as carbidopa and benserazide, which cannot enter the brain to interfere with the conversion of levodopa in the brain. Collectively, inhibition of the peripheral AADC could increase the bioavailability of levodopa.

Moreover, COMT could convert levodopa into catecholamines, such as epinephrine, which further causes arrhythmia. Therefore, the clinical use of levodopa also combines with COMT inhibitors, such as entacapone and tolcapone, to enhance the efficacy of levodopa. However, strict attention should be paid to the patient's liver function upon tolcapone administration [17]. However, when COMT inhibitors are used as adjuvant therapy for PD, the life quality of patients is not as good as dopamine agonists [18].

### 2.2 Metabolism of Levodopa by Gut Bacteria

#### 2.2.1 Enterococcus faecalis (E. faecalis)

Pyridoxal phosphate-dependent bacterial tyrosine decarboxylase (TDC) is involved in the decarboxylation of both tyrosine and levodopa [19]. Several species of the genus *Lactobacillus* and *Enterococcus* have been discerned to contain TDC [20, 21]. To determine whether the genomes of other gut bacteria also encode TDC, the TDC protein sequence of *E. faecalis* v583 (EOT87933) was used *via* the US National Institutes of Health Human Microbiota Project (HMP) protein database. This analysis identified TDC proteins only in species belonging to the bacillus class, including over 50 *Enterococcus* strains (mainly *E. faecium* and *E. faecalis*) and several *Lactobacillus* and *Staphylococcus* species. We compared the genome of *E. faecalis* v583 with two gut bacteria isolates, *E. faecium* W54 and *L. brevis* W63, and their TDC operon genes turned out to be perfectly aligned, illustrating the conservation of TDC operon among these species [19]. Moreover, *E. faecium* and *E. faecalis* were found to convert levodopa to dopamine more efficiently than *L. brevis* [19].

Recent studies confirm that TDC could metabolize levodopa and convert tyrosine. Tyrosine is the preferred substrate for TDC and exists in the small intestine [22, 23]. However, the concentration of tyrosine does not affect levodopa decarboxylation. Once excess tyrosine is present, levodopa and tyrosine are converted simultaneously [19]. Similarly, human AADC inhibitors do not inhibit bacterial decarboxylase. None of the three common human AADC inhibitors, carbidopa, benserazide and methyldopa, inhibit levodopa decarboxylase activity in *E. faecalis* and *E. faecium* [19].

Since TDC gene sequences are highly conserved and *E. faecalis* is the main microorganism responsible for levodopa decarboxylation, a strong linear relationship has been found between TDC and *E. faecalis* abundance. Thus, differences in the metabolism of levodopa among individuals could be predicted by the abundance of TDC gene in *E. faecalis* [24]. Further, once the abundance of TDC genes in PD patients is higher, the bioavailability of levodopa apparently becomes lower. The main reason for this is that bacterial TDC converts part of levodopa into dopamine, reducing its therapeutic effect, and thus increasing the dose of levodopa and triggering drug side effects. The proximal part of the small intestine is the main zone of levodopa absorption. Current studies indicate that the abundance of TDC in this region of rats is negatively correlated with the levodopa content in the blood [19].

#### 2.2.2 Eggerthella lenta (E. lenta)

After identifying an intestinal bacterium levodopa decarboxylase, the conversion of dopamine to m-tyramine was next examined. This activity might affect peripheral levodopa decarboxylase activity, which could be associated with the adverse reactions of the peripheral metabolism of levodopa [25]. It was shown that *E. faecalis* did not metabolize dopamine further, and the next step was performed by other bacteria. One was the bacterium *E. lenta.* This strain was able to selectively remove the p-hydroxyl group of dopamine to produce m-tyramine through its production of the molybdenum cofactor-dependent dopamine dehydroxylase (Dadh) enzyme. This was the first record of a bacterium that dehydroxylated dopamine.

A basic local alignment search tool P (BLASTP) study showed that the gene for this molybdenum-dependent dehydroxylase was restricted to *E. lenta* and its close actinobacterial relatives, which led to the screening of 26 enteric actinomycete isolates for their ability to dehydroxylate dopamine in anaerobic culture [26]. The results demonstrated that only 10 Eggerthella strains could convert dopamine to m-tyramine. This strain-level variability in the metabolism of dopamine strengthened the character of the gut microbial species and did not predict the metabolism of dopamine [26, 27]. A single-nucleotide polymorphism (SNP) has been found in the gene for metabolizing *versus* non-metabolizing enzymes. For example, in the gene for the strain that metabolizes dopamine, position 506 is arginine, while in the inactive strain, it is serine. It is believed that specific amino acid residues in the Dadh enzyme are applied to predict dopamine dehydrogenation [24].

**Fig. (1):**
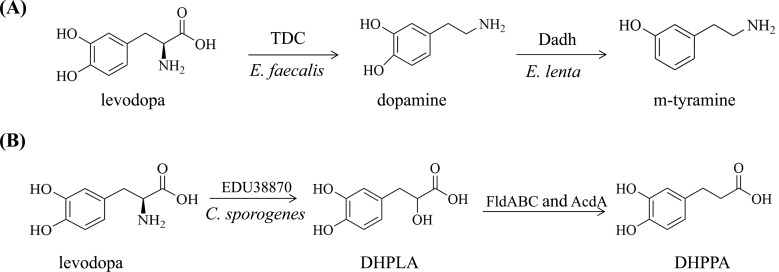
Metabolism of levodopa by gut bacteria. (**A**) Proposed major pathway participated in levodopa metabolism by *E. faecalis* and *E. lenta.* (**B**) *C. sporogenes* was able to deaminate levodopa *via* the deamination pathway under anaerobic conditions.

The two bacteria described above exhibited a complete levodopa metabolic pathway that existed among intestinal bacterial species (Fig. **1A**). Firstly, pyridoxal phosphate-dependent TDC produced by *E. faecalis* converted levodopa to dopamine, and then molybdenum-dependent dehydroxylase produced by *E. lenta* converted dopamine to m-tyramine. The activities of both bacterial-produced enzymes were involved in the metabolism of levodopa, and both enzymes predicted levodopa metabolism at different molecular levels.

#### 2.2.3 Clostridium sporogenes (C. sporogenes)

In addition to *E. faecalis* and *E. lenta*, levodopa can also be metabolized by deamidation to produce 3-(3, 4-dihydroxyphenyl) propionic acid (DHPPA) [28]. There are many different sources of aromatic amino acids in the intestine [29]. Bacteria in the intestine can metabolize aromatic amino acids. In the lower part of the gastrointestinal tract, where oxygen is limited, anaerobic bacterial degradation of aromatic amino acids involves reduction or oxidative deamination [30], resulting in the production of aromatic metabolites [31-34]. These metabolites in the human body are closely associated with intestinal barrier action, immune regulation and intestinal motility functions [9, 35-38]. Early *in vivo* studies showed that approximately 90% of levodopa was delivered to the circulatory system [39-41], leaving an unabsorbed residual levodopa fraction of approximately 10% that served as a substrate for other bacteria associated with the lower and more anaerobic regions of the gastrointestinal tract [42]. Also, levodopa underwent deamination in the same way as aromatic amino acids [28].

*C. sporogenes* is able to deaminate protein-derived aromatic amino acids (PAAAs) *via* the deamination pathway under anaerobic conditions [34, 43, 44]. It was demonstrated that *C. sporogenes* also deaminated levodopa through the deamination pathway [28]. In detail, levodopa was used as a substrate with other aromatic amino acids to initiate the deamination process by the action of aromatic amino acid transaminase (AAT), such as EDU38870, produced by *C. sporogenes*. Then, AAT metabolized levodopa to the intermediate 3-(3, 4-dihydroxyphenyl) lactic acid (DHPLA), which was completely metabolized by dehydrogenases (FldH and AcdA) and dehydratase (FldABC) to the end product DHPPA (Fig. **1B**). During this process, AAT was the key enzyme that initiated the deamination pathway of levodopa. Furtherly, a positive correlation between bacterial-derived polyphenol metabolites and human intestinal transit time was exhibited [45]. As a phenolic acid (a type of molecule in polyphenols), DHPPA had an obvious inhibitory action on ileal muscle activity after acetylcholine stimulation in mice [28]. In the faeces of PD patients treated with levodopa, the content of DHPPA metabolized by gut microbiota was higher than that in healthy controls. It was demonstrated that DHPPA was produced by microbiota *in vivo* through anaerobic deamination of levodopa. DHPPA could be further converted by bacteria to 3-(3-hydroxyphenyl) propionic acid (3-HPPA) *in vitro*. This conversion was determined by *E. lenta*, which was previously reported to present its ability to parahydroxylate DHPPA [46, 47]. Then, whether 3-HPPA could exert a similar response to acetylcholine-induced ileal contractions was examined. In contrast to DHPPA, 3-HPPA had no remarkable effect on acetylcholine-induced twitching [28].

Gastrointestinal dysfunction occurs frequently in patients with PD. Most notably, constipation is observed in 80-90% of patients with PD [48]. Of importance is that chronic idiopathic constipation is related to motor abnormalities [49, 50]. Studies have shown that DHPPA can be detected in 70% of PD samples by measuring the product of DHPPA or 3-HPPA in the stool of PD patients, demonstrating that the aromatic deamination pathway is active in PD patients. DHPPA inhibits acetylcholine-induced ileal muscle contraction and further affects gut motility, which might exacerbate intestinal function in patients. Currently, *C. sporogenes* was shown to effectively deaminate unabsorbed levodopa residues to DHPPA in the feces of PD patients, thereby decreasing isolated ileal motility. Collectively, the potential effects of the absorption of drugs by the intestine require greater attention and prevention.

#### 2.2.4 Other Bacteria

In addition to metabolizing levodopa, recent studies suggest that gut microbiota can increase levodopa synthesis under pharmacological modulation and further improve brain function. The main synthesis pathway of dopamine in humans is “L-phenylalanine (Phe) → L-tyrosine (Tyr) → (S)-3, 4-dihydroxyphenylalanine (levodopa) → dopamine” [51, 52]. The first step in dopamine synthesis is the absorption of tyrosine into the brain. Food proteins are rich in tyrosine. In addition, phenylalanine contained in food can be converted to tyrosine by the action of phenylalanine hydroxylase (PAH) in the liver and kidneys, and also in dopamine neurons by tyrosine hydroxylase (TH). Both phenylalanine-converted and food-derived tyrosine in the blood enters dopamine neurons in the brain *via* amino acid transporters. Next, it is metabolized to levodopa by the enzyme TH in the cytoplasm of dopamine neurons. This step is the rate-limiting step in the whole dopamine synthesis process regulated by TH. The third step is the decarboxylation of levodopa to dopamine by the action of AADC. The coenzyme of TH is tetrahydrofolate (BH_4_) [53], which acts as an electron carrier in the enzymatic reaction as a reducing agent. Its oxidized form is dihydrobiopterin, which is regenerated into tetrahydrobiopterin by reduction with NADPH as the hydrogen donor catalyzed by dihydrobiopterin reductase (DHPR), and the coenzyme of AADC is 5'-phosphate (PLP) [54].

Moreover, berberine (BBR) is a natural compound extracted from herbs, such as *Coptis chinensis* and *Berberis vulgaris.* In China, BBR has been used for decades as a treatment for diarrhea. Previous studies investigated whether BBR ameliorated PD, in which one group found the oral administration of BBR as effective in treating the PD mouse model [55], but the other group obtained negative results with an intraperitoneal injection of BBR in the PD rat model [56]. Thus, it was assumed that gut microbiota might be the answer. Then, the effects of BBR on 10 intestinal bacterial strains were further determined [57]. Within these 10 strains, four bacterial strains (*E. faecalis*, *E. faecium*, *Proteus mirabilis* and *Lactobacillus acidophilus*) exhibited an obvious increase in dopamine levels after BBR treatment. Based on dopamine production, *E. faecalis* and *E. faecium* were selected for further investigation.

**Fig. (2):**
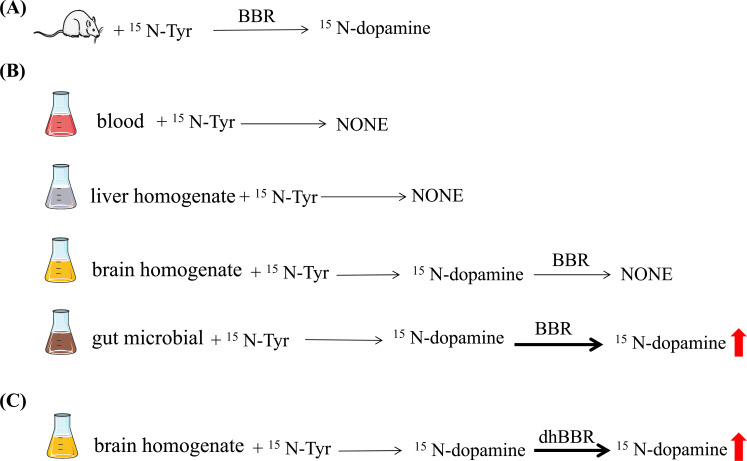
The increased brain dopamine level by BBR might be derived from dopamine in the gut microbiota.

To verify the action of BBR on the gut-brain axis, *E. faecalis* was introduced into the gut of PD mice, and this bacterium apparently increased brain dopamine levels and improved the motor performance of PD mice. In an *in vivo* experiment, ^15^N-tyrosine was injected into the colon of mice, and then BBR was orally administered. As a result, ^15^N-dopamine was well detected in the mouse brain (Fig. **2A**). To further identify whether the gut microbiota might be a source of brain dopamine supply in the body, the blood, liver homogenate, brain homogenate or gut microbiota from PD mice were cultured *in vitro* with the isotopically labeled ^15^N-tyrosine, respectively. Results indicated that ^15^N-labeled dopamine was discerned in brain homogenates and intestinal bacteria, whereas it was not detected in blood and liver homogenates, demonstrating brain and intestinal bacteria to be the main sites of dopamine biosynthesis *in vivo*.

Furthermore, when BBR was added to the brain and intestinal tissue in *in vitro* experiments, ^15^N-labeled dopaminewas increased in intestinal bacteria but not in brain tissue(Fig. **2B**). This was because brain tissue did not contain dihydroberberine (dhBBR). Combined with *in vivo* experiments described above, it was suggested that the increased brain dopamine content by BBR might be derived from dopamine in gut microbiota, which could be an alternative dopamine-producing “organ” besides the brain.

Additionally, the addition of BBR to the brain homogenate did not increase the level of levodopa/dopamine in the brain. However, co-incubation of brain homogenate with dhBBR, increased the levodopa/dopamine level in the brain (Fig. **2C**). The dhBBR was that BBR metabolite produced by nitroreductase in gut microbiota and dhBBR was detected only in the intestine [58]. This might provide strong evidence for this hypothesis that the increased dopamine in the brain by BBR was from the gut microbiota. Meanwhile, the activity of TH and the co-enzyme BH_4_ and the level of AADC and the co-enzyme PLP in the brain were elevated by dhBBR. Thus, BBR-induced increase of levodopa/dopamine production in the gut microbiota might be mediated through its intestinal metabolite dhBBR, which could activate TH and AADC by elevating BH_4_ and PLP activities, respectively.

Additional studies show that BBR activates TH and AADC enzyme activities in *E. faecalis* and *E. faecium* bacteria to increase levodopa/dopamine synthesis. TH consumes BH_4_ in the production of levodopa and converts it to 7, 8-dihydrobiopterin (BH_2_), in which BH_4_ is continuously consumed. BH_4_ can be synthesized in the cytoplasm in two ways [59]. In detail, under normal conditions, BH_4_ can be synthesized from the GTP (guanosine triphosphate)-NH_2_P_3_ (D-erythro-7,8-dihydroneopterin triphosphate) - PPH_4_(6-pyruvoyltetra-hydropterin) - BH_4_ pathway, which is called “source synthesis”; on the other hand, BH_4_ can be replenished by means of a remedial pathway of sepiapterin →BH_2_→BH_4_, and dihydrofolate reductase (DHFR) is the critical enzyme responsible for transforming BH_2_ to BH_4_ [59].

**Fig. (3):**
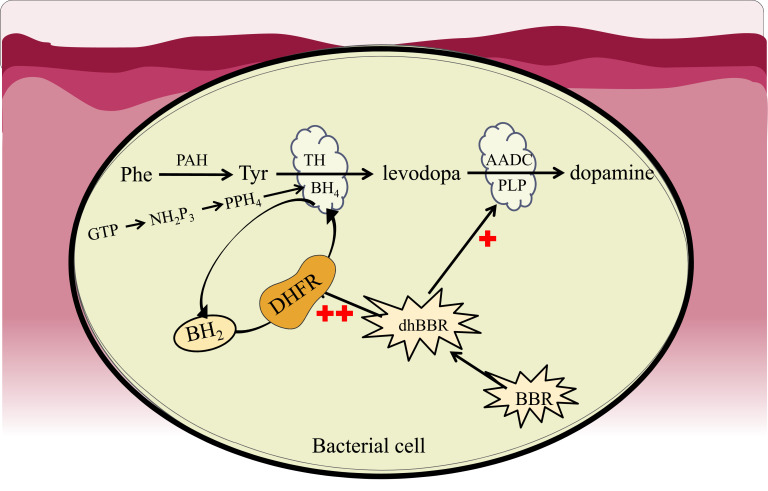
Regulation of dopamine biosynthesis in the gut microbiota by BBR. **Abbreviations**: Phe: L-phenylalanine; PAH: phenylalanine hydroxylase; Tyr: L-tyrosine; TH: tyrosine hydroxylase; BH_4_: The coenzyme of TH is tetrahydrofolate; BH_2_: 7, 8-dihydrobiopterin; DHFR: dihydrofolate reductase; BBR: berberine; dhBBR: dihydroberberine; GTP: guanosine triphosphate; PPH_4_: 6-pyruvoyltetrahydropterin; NH_2_P_3_: D-erythro-7, 8-dihydroneopterin triphosphate.

After oral administration of BBR, dhBBR produced by bacterial nitroreductase could provide H ions with a function similar to that of the co-enzyme NADPH [58]. Indeed, dhBBR is able to increase the biological activity of DHFR to promote the biotransformation of BH_2_ to BH_4_. Thus, dhBBR appears to be an activator of DHFR (Fig. **3**). Moreover, BBR enhances TH activity by increasing the activity of DHFR, which promotes the production of BH_4_ from BH_2_, thereby accelerating the generation of levodopa by gut bacteria.

It has been found that in addition to *E. faecalis,* there are multiple approaches that other bacteria employ to accomplish the action of TH and AADC. By searching the NCBI database, *Ralstonia solanacearu* and *Streptomyces achromogenes* were found to contain homologous genes for TH [57], and tyrosinases in *Pseudomonas* and *Bacillus* could transform tyrosine into levodopa [60-62]. Further, two protein sequences of hydroxylase and ten protein sequences of decarboxylases in *E. faecalis* were detected. These results suggest that intestinal bacteria could directly synthesize dopamine. A summary of some of the gut bacteria influenced levodopa metabolism is given in Table **1**.

## 3 FACTORS INVOLVED IN THE REDUCED METABOLISM OF LEVODOPA

### 3.1 AADC Inhibitors, COMT Inhibitors, Dopamine Receptor Agonists and Monoamine Oxidase (MAO) Inhibitors

As mentioned earlier, the main synthetic pathway of dopamine in the body refers to “Phe → Tyr → levodopa → dopamine” metabolic pathway. Disruption of this pathway leads to various neurological disorders, such as PD and phenylketonuria [63].

**Fig. (4):**
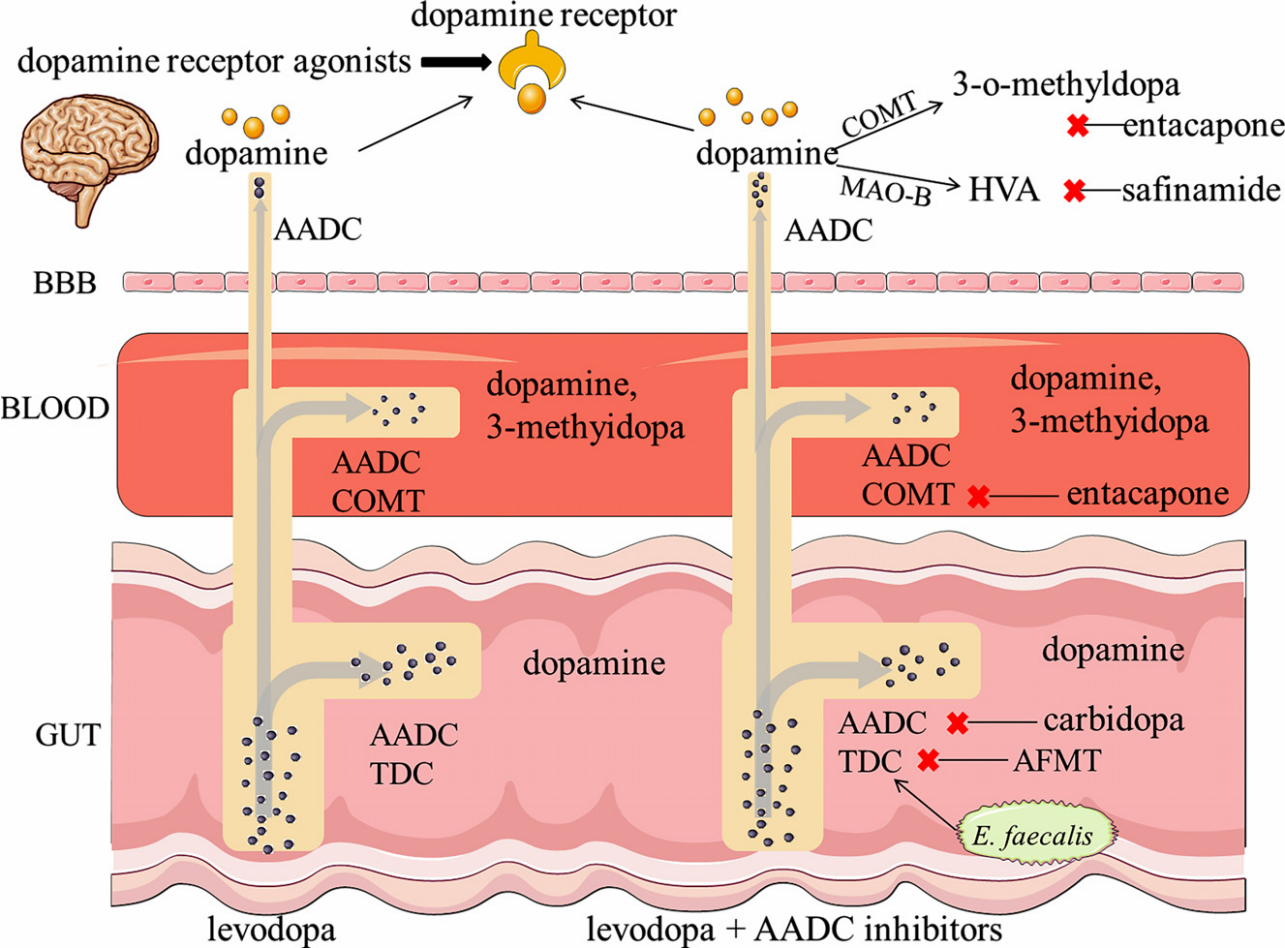
Metabolism of levodopa and the involved bacterial enzymes indicated together with their specific inhibitors. **Abbreviations**: BBB: blood-brain barrier; AADC: aromatic amino acid decarboxylase; COMT: catechol-O-methyltransferase; AFMT: (S)-α-fluoromethyltyrosine; MAO-B: monoamine oxidase-B; HVA: homovanillic acid.

The first-line clinical treatment of PD is oral levodopa. Levodopa crosses BBB and can be directly converted to dopamine by human AADC once it enters the neuron. The rate-limiting enzyme step of converting tyrosine to levodopa is skipped, thus allowing rapid replenishment of dopamine in the striatum. However, more than 95% of orally administered levodopa is metabolized to dopamine in the periphery by AADC, and only 1% to 5% crosses BBB into the brain as the drug prototype [5, 64, 65]. Levodopa is metabolized in the periphery in two main ways [66]. One is that most levodopa is converted to dopamine by AADC. Dopamine cannot cross BBB and is eventually metabolized to homovanillic acid (HVA) and dihydroxyphenylacetic acid (DOPAC). Increased peripheral dopamine might have negative effects on the body, most commonly causing nausea and vomiting [25, 67]. The other is metabolized to 3-o-methyldopa by COMT (Fig. **4**). To reduce the metabolism of levodopa in the periphery, more levodopa enters the brain to be converted to dopamine for action; levodopa is often combined with AADC inhibitors, such as carbidopa or benserazide. The reason is that carbidopa or benserazide could effectively inhibit AADC activity by forming a stable covalent hydrazine linkage with the tyrosine decarboxylase cofactor PLP [68]. Nevertheless, AADC inhibitors cannot cross BBB and have no effects on the decarboxylation of levodopa in the brain [68, 69]. Thus, AADC inhibitors could reduce the metabolism of levodopa in the periphery, then indirectly increase dopamine content in the brain, and ultimately improve PD symptoms. In addition, the combination of COMT inhibitors with levodopa also reduces the metabolism of levodopa in the periphery. Tolcapone and entacapone are common COMT inhibitors [70]. Tolcapone is less commonly used due to its acute side effects of liver damage. Entacapone only inhibits peripheral COMT, which is milder and has a better safety profile than tolcapone [71]. In summary, neither AADC inhibitors nor COMT inhibitors can be used alone in the treatment of PD. They need to be combined with levodopa to increase the bioavailability of levodopa, and thus improve the symptoms of PD patients.

In addition, there are other often prescribed pharmaceuticals, like dopamine receptor agonists and monoamine oxidase (MAO) inhibitors. Dopamine receptor agonists include the ergot derivatives, such as bromocriptine, cabergoline and pergolide, and the non-ergot derivatives, such as piribedil, pramipexole and ropinirole. These agonists modulate the activity of dopamine receptors in the brain, making the dopamine receptors in the brain more sensitive to dopamine [72]. Moreover, dopamine receptor agonists can be used either alone in the PD early stage or in combination with levodopa in PD later stage [73]. To some extent, this combination reduces the dose of levodopa, and thus decreases the occurrence of the adverse effects induced by high-dose levodopa. On the other hand, MAO-B plays an important role in the dopamine metabolic pathway. It breaks down dopamine into HVA, accompanied by the generation of the free radical H_2_O_2_, which has neurotoxic effects [74]. MAO-B inhibitors might have a dual effect, including reducing the catabolism of dopamine and limiting the formation of neurotoxic free radicals. As a new potent and highly specific inhibitor of MAO-B, safinamide results in an increase in the bioavailability of levodopa to further enhance the duration of levodopa effects on PD [70]. The roles of several of the above compounds in levodopa metabolism are summarized in Table **2**.

### 3.2 Antibiotics

A growing number of studies have shown that gut microbiota interferes with the therapeutic effects of levodopa. Antibiotic treatment was proven to reduce the intestinal metabolism of levodopa, resulting in improvements in the efficacy of levodopa. By studying the correlation between gut microbes and the efficacy of levodopa, it was found that *E. faecalis* abundance was higher in the gut of patients with drug failure than in those with drug efficacy. *E. faecalis* produces bacterial TDC, which metabolizes levodopa into dopamine. Further, the newly generated dopamine is degraded into smaller molecules, and finally, levodopa is completely metabolized.

Recent clinical studies have shown that *Helicobacter pylori* (*H. pylori*) infection is indeed more prevalent in patients with PD compared to controls [75-78]. It was further confirmed that *H. pylori* infection had a negative effect on motor performance in PD patients and that stride length was improved after *H. pylori* eradication [79]. Most likely, *H. pylori* infection might affect the absorption of levodopa through multiple mechanisms. For example, *H. pylori* can alter the pH of gastric acid [80-83], disrupt gastric motility, and prolong gastric emptying time [84, 85]. These factors may act synergistically to impair the intestinal absorption of levodopa *in vivo*. Additional evidence indicated that PD patients with *H. pylori* eradication presented an apparent improvement in their clinical symptoms as well as an obvious increase in their plasma levodopa level when compared to controls without *H. pylori* eradication [7]. In clinical practice, *H. pylori* is treated with bismuth and proton pump inhibitors or a combination of these two antibiotics as the main treatment [86, 87]. Also, tetracycline and metronidazole are the more commonly used antibiotics to treat *H. pylori*.

Furthermore, small intestinal bacterial overgrowth (SIBO) is a malabsorption syndrome associated with increased bacterial density above 10^5^ colony-forming units/mL of small intestinal aspirate and/or the presence of colonic-type species [88]. Previous studies indicate abnormally slow intestinal motility as an established susceptibility factor for the development of SIBO [88]. Since constipation and bloating in PD patients are clinically associated with slow intestinal motility, gastrointestinal dysfunction seemed to be the most likely pathophysiological link between SIBO and PD. Besides, PD patients had a higher chance of suffering from SIBO than normal controls [89]. SIBO caused levodopa to be poorly absorbed in the intestine and also LID. Levodopa metabolism and LID were improved when SIBO was treated [6]. Further, SIBO impairs the absorption of levodopa in a multifactorial manner. First, loss of brush border disaccharidase activity due to mucosal damage, bacterial fermentation of sugars, and bacterial breakdown of bile acids might lead to malabsorption [90, 91]. Second, SIBO could affect the absorption of levodopa in the jejunum upon intestinal mucosal inflammation [88, 90-92].

Interestingly, studies have confirmed that the prevalence of *H. pylori* infection in patients with PD is not higher than that in healthy controls [78, 93]. However, *H. pylori* infection plays a synergistic role with SIBO in the pathogenesis of motor fluctuations and affects levodopa metabolism [6]. Therefore, both infections of *H. pylori* and SIBO should be considered during PD treatment. Levodopa efficacy could be improved by appropriate antibiotic medication. However, attention should be paid to the recurrence of SIBO.

The wide-spectrum antibiotics, such as metronidazole and ciprofloxacin, are commonly used to treat *H. pylori* and SIBO [88]. There is no evidence that antibiotics alone are effective in blocking levodopa conversion in the gut. Actually, antibiotics directly affect gut bacteria to further indirectly influence levodopa metabolism. It has also been documented that two-thirds of currently existing drugs are affected by intestinal bacteria [94]. So, the effect of antibiotics on levodopa is actually the effect of antibiotics on the TDC-producing bacteria in the intestine. In addition, previous studies used genetics and *in vitro* biochemistry experiments to confirm that TDC was necessary and sufficient for levodopa decarboxylation by *E. faecalis* [24]. *E. faecalis* MMH594 mutants carrying a 2-kb Tetcassette disrupted TDC so that TDC could not decarboxylate levodopa. Importantly, no apparent difference was observed in growth between *E. faecalis* MMH594 mutant mice and wild-type mice [24].

Taken together, levodopa is metabolized in the intestinal tract due to the action of bacteria, which further reduces levodopa bioavailability. Therefore, appropriate antibiotics can be applied upon PD treatment to reduce the interference of *E. faecalis*, *H. pylori* and other intestinal bacteria. It has also been shown that intestinal worm parasites affect levodopa metabolism and then improve the bioavailability of levodopa after antibiotic therapy. For example, a PD patient presented levodopa malabsorption caused by *Strongyloides stercoralis* duodenitis. After the eradication of the parasitic disease, the motor fluctuations were greatly improved and also a 33% reduction in levodopa dose was achieved [95].

### 3.3 Low pH

It was shown that at lower pH environments, the acidic environment caused an increase in TDC transcription and a correspondingly more active conversion of tyrosine [21]. The reason is that during the decarboxylation of tyrosine to produce tyramine, *E. faecalis* might generate a mechanism to maintain the cytoplasmic pH that helps cope with an acidic environment, further resulting in more survival of *E. faecalis*. However, the specific mechanism by which tyrosine decarboxylation functions in tolerance to acidity is not clear. The present data showed that the decarboxylation reaction was able to neutralize acidification in the cell, while the degree of decarboxylation was dependent on the external pH value [96]. It was confirmed that bacterial conversion of levodopa was accelerated at a lower pH value. Moreover, the environment of different parts of the intestines was not the same, and the pH value was not identical either [97]. Thus, the levodopa decarboxylation reaction might be accelerated in the small intestine with a lower pH value [92]. Also, this could be speculated that proper improvement of the intestinal pH environment would reduce the metabolism of levodopa.

### 3.4 AFMT

Carbidopa is a levodopa analogue that inhibits AADC by forming a stable covalent hydrazone bond with the tyrosine decarboxylase cofactor PLP [68]. Based on the working principle of carbidopa, it was found that ɑ-fluoromethylamino acid (AFMT), which was an L-tyrosine analogue, selectively inhibited the production of TDC by intestinal microbiota. Investigation of potential amino acid substrates has revealed that TDC requires a p-hydroxyl group for powerful activity, whereas AADC prefers the m-hydroxyl substituent. Therefore, it is hypothesized that the L-tyrosine analogue, AFMT, might selectively inhibit TDC. This means that AFMT is able to only inhibit the effect of intestinal bacteria on levodopa without affecting the normal decarboxylation of levodopa in the central nervous system. In addition, *in vitro* studies demonstrated that AFMT inhibited levodopa decarboxylation of TDC but not AADC, suggesting that AFMT could form PLP adducts with TDC and inhibit TDC enzyme activity. *In vivo* studies exhibited that AFMT effectively reduced the metabolism of levodopa by intestinal microorganisms in the organism and thus increased levodopa bioavailability in mice [24].

## CONCLUSION

Current treatment of PD mainly relies on levodopa to relieve the motor disorder, although levodopa could induce dyskinesia and symptom fluctuations. Reducing the fluctuation of levodopa levels and extending the half-life are the requirements for levodopa today. Until now, levodopa is being applied in combination with drugs, such as AADC inhibitors, to inhibit the levodopa metabolic pathway. There is increasing evidence that gut microbiota plays an important role in the metabolism of levodopa and the synthesis of levodopa from L-tyrosine by gut bacteria. To improve the bioavailability of levodopa, the mechanism of action of levodopa in intestinal bacteria warrants further elucidation. Thus, changing the gut microbiome and understanding the metabolic pattern of levodopa in intestinal microorganisms might open a new promising avenue in future drug development and PD prevention and treatment.

## Figures and Tables

**Table 1:** Gut bacteria influencing levodopa metabolism.

**Gut Bacteria**	**Function**	**References**
*E. faecalis*	Encode TDC and convert levodopa into dopamine.	Kessel *et al*. [19] Rekdal *et al*. [24]
*E. lenta*	Metabolize dopamine to produce m-tyramine through its production of Dadh.	
*C. sporogenes*	Metabolize levodopa by deamidation to produce DHPPA.	Kessel *et al*. [28]
Other bacteria (*Proteus mirabilis* and *Lactobacillus acidophilus*)	Increase dopamine content after BBR treatment.	Wang *et al*. [57]

**Table 2:** AADC inhibitors, COMT inhibitors, dopamine receptor agonists and monoamine oxidase (MAO) inhibitors influencing levodopa metabolism.

**Compounds**	**Function**	**References**
AADC inhibitors (carbidopa and benserazide)	Block the metabolism of levodopa to dopamine by AADC.	Burkhard *et al.* [68]
COMT inhibitors (entacapone)	Block the metabolism of levodopa to 3-o-methyldopa by COMT.	Fabbri *et al.* [71]
Dopamine receptor agonists (piribedil and pramipexole)	Increase dopamine receptor activity in the brain.	Latt *et al.* [73]
MAO-B inhibitors (safinamide)	Reduce the catabolism of dopamine and limit the formation of neurotoxic free radicals.	Tan *et al.* [74]

## LIST OF ABBREVIATIONS

AADC: Amino Acid Decarboxylase
BBB: Blood-Brain Barrier
HVA: Homovanillic Acid
LID: Levodopa-Induced Dyskinesia
PAAAs: Protein-Derived Aromatic Amino Acids
PAH: Phenylalanine Hydroxylase
PD: Parkinson's Disease
TH: Tyrosine Hydroxylase

## References

[1] Ahlskog J.E., Muenter M.D. (2001). Frequency of levodopa‐related dyskinesias and motor fluctuations as estimated from the cumulative literature.. Mov. Disord..

[2] Fahn S. (2003). Description of Parkinson’s disease as a clinical syndrome.. Ann. N. Y. Acad. Sci..

[3] Manson A., Stirpe P., Schrag A. (2012). Levodopa-induced-dyskinesias clinical features, incidence, risk factors, management and impact on quality of life.. J. Parkinsons Dis..

[4] Perez-Lloret S., Negre-Pages L., Damier P., Delval A., Derkinderen P., Destée A., Meissner W.G., Tison F., Rascol O. (2017). LDOPA-induced dyskinesias, motor fluctuations and health-related quality of life: the COPARK survey.. Eur. J. Neurol..

[5] Papavasiliou P.S., Cotzias G.C., Düby S.E., Steck A.J., Fehling C., Bell M.A. (1972). Levodopa in Parkinsonism: potentiation of central effects with a peripheral inhibitor.. N. Engl. J. Med..

[6] Fasano A., Bove F., Gabrielli M., Petracca M., Zocco M.A., Ragazzoni E., Barbaro F., Piano C., Fortuna S., Tortora A., Di Giacopo R., Campanale M., Gigante G., Lauritano E.C., Navarra P., Marconi S., Gasbarrini A., Bentivoglio A.R. (2013). The role of small intestinal bacterial overgrowth in Parkinson’s disease.. Mov. Disord..

[7] Hashim H., Azmin S., Razlan H., Yahya N.W., Tan H.J., Manaf M.R.A., Ibrahim N.M. (2014). Eradication of Helicobacter pylori infection improves levodopa action, clinical symptoms and quality of life in patients with Parkinson’s disease.. PLoS One.

[8] Qin J., Li R., Raes J., Arumugam M., Burgdorf K.S., Manichanh C., Nielsen T., Pons N., Levenez F., Yamada T., Mende D.R., Li J., Xu J., Li S., Li D., Cao J., Wang B., Liang H., Zheng H., Xie Y., Tap J., Lepage P., Bertalan M., Batto J.M., Hansen T., Le Paslier D., Linneberg A., Nielsen H.B., Pelletier E., Renault P., Sicheritz-Ponten T., Turner K., Zhu H., Yu C., Li S., Jian M., Zhou Y., Li Y., Zhang X., Li S., Qin N., Yang H., Wang J., Brunak S., Doré J., Guarner F., Kristiansen K., Pedersen O., Parkhill J., Weissenbach J., Bork P., Ehrlich S.D., Wang J. (2010). A human gut microbial gene catalogue established by metagenomic sequencing.. Nature.

[9] Yano J.M., Yu K., Donaldson G.P., Shastri G.G., Ann P., Ma L., Nagler C.R., Ismagilov R.F., Mazmanian S.K., Hsiao E.Y. (2015). Indigenous bacteria from the gut microbiota regulate host serotonin biosynthesis.. Cell.

[10] Mao K., Baptista A.P., Tamoutounour S., Zhuang L., Bouladoux N., Martins A.J., Huang Y., Gerner M.Y., Belkaid Y., Germain R.N. (2018). Innate and adaptive lymphocytes sequentially shape the gut microbiota and lipid metabolism.. Nature.

[11] Pusceddu M.M., El Aidy S., Crispie F., O’Sullivan O., Cotter P., Stanton C., Kelly P., Cryan J.F., Dinan T.G. (2015). N-3 Polyunsaturated Fatty Acids (PUFAs) Reverse the Impact of Early-Life Stress on the Gut Microbiota.. PLoS One.

[12] El Aidy S., van Baarlen P., Derrien M., Lindenbergh-Kortleve D.J., Hooiveld G., Levenez F., Doré J., Dekker J., Samsom J.N., Nieuwenhuis E.E.S., Kleerebezem M. (2012). Temporal and spatial interplay of microbiota and intestinal mucosa drive establishment of immune homeostasis in conventionalized mice.. Mucosal Immunol..

[13] Kelly J.R., Borre Y., O’ Brien C., Patterson E., El Aidy S., Deane J., Kennedy P.J., Beers S., Scott K., Moloney G., Hoban A.E., Scott L., Fitzgerald P., Ross P., Stanton C., Clarke G., Cryan J.F., Dinan T.G. (2016). Transferring the blues: Depression-associated gut microbiota induces neurobehavioural changes in the rat.. J. Psychiatr. Res..

[14] Niehues M., Hensel A. (2009). In-vitro interaction of L-dopa with bacterial adhesins of Helicobacter pylori: an explanation for clinicial differences in bioavailability?. J. Pharm. Pharmacol..

[15] Enright E.F., Gahan C.G., Joyce S.A., Griffin B.T. (2016). The impact of the gut microbiota on drug metabolism and clinical outcome.. Yale J. Biol. Med..

[16] Hsu A., Yao H.M., Gupta S., Modi N.B. (2015). Comparison of the pharmacokinetics of an oral extended‐release capsule formulation of carbidopa‐levodopa (IPX066) with immediate‐release carbidopa‐levodopa (Sinemet®), sustained‐release carbidopa‐levodopa (Sinemet® CR), and carbidopa‐levodopa‐entacapone (Stalevo®).. J. Clin. Pharmacol..

[17] Longo D.M., Yang Y., Watkins P.B., Howell B.A., Siler S.Q. (2016). Elucidating differences in the hepatotoxic potential of tolcapone and entacapone with DILIsym®, a mechanistic model of drug‐induced liver injury.. CPT Pharmacometrics Syst. Pharmacol..

[18] Gray R., Patel S., Ives N., Rick C., Woolley R., Muzerengi S., Gray A., Jenkinson C., McIntosh E., Wheatley K., Williams A., Clarke C.E., Young K., Price H., Price J., Lambert A., Reeve R., Sewell M., Broome S., Williams A., Baker M., Clarke C., Fitzpatrick R., Gray A., Greenhall R., Jenkinson C., Mant D., McIntosh E., Sandercock P., Baugent C., Crome P., Au P., Boodell T., Cheed V.C., Daniels J., Dowling F., Evans L., Hawker R., Kaur S., Rick C., Wheatley K., Winkles N., Hingley D., Sturdy L., Wooley R., Ottridge R., Peto L., Hilken N., Counsell C., Caie L., Caslake R., Coleman R., Crowley P., Gerrie L., Gordon J., Harris C., Leslie V., MacLeod M.A., Taylor K., Worth P., Barker R.A., Forsyth D., Halls M., Young J., Phillips W., Manford M., Thangarajah N., Blake D., Prescott R., Carr P., Cochrane L., Rose A., McLaren A., Drover M., Karunaratne P., Eady A., Wislocka-Kryjak M., Ghaus N., Grueger A., Mallinson B., Wihl G., Ballantyne S., Hutchinson S., Lewthwaite A., Nicholl D., Ritch A., Coyle S., Hornabrook R., Irfan H., Poxon S., Nath U., Davison J., Dodds S., Robinson G., Gray C., Fletcher P., Morrow P., Sliva M., Folkes E., Gilbert A., Hayes H., Burrows E., Donaldson S., Lawrence J., Rhind G., Baxter G., Bell J., Gorman J., Guptha S., Noble C., Hindle J., Jones S., Ohri P., Subashchandran R., Roberts E., Raw J., Wadhwa U., Aspden L., Partington L., Vanek H., Whone A., Barber R., Haywood B., Heywood P., Lewis H., O’Sullivan K., Prout K., Whelan L., Medcalf P., Sliva M., Fuller G., Morrish P., Wales E., Dalziel J., Overstall P., Bouifraden K., Evans C., Ward G., Matheson P., Lockington T., Graham A., Grimmer S.F.M., Sheehan L.J., Williams H., Hubbard I., Walters R., Glasspool R., Critchley P., Abbott R., Kendall B., Lawden M., Lo N., Rajaally Y., Simpson B., Martey J., Wray L.G., Omar M., Sharma A., Gale A., Phirii D., Sekaran L., Wijayasiri S., Silverdale M., Walker D., Fleary H., Monaghan A., Senthil V., Reynolds S., Chong M.S., Diem D., Kundu B., Arnold D., Quinn N., Benamer H., Billings J., Corston R., D’Costa D., Green M., Shuri J., Noble J.M., Cassidy T., Gani A., Lawson R., Nirubin A., Cochius J., Dick D., Lee M., Payne B., Roche M., Sabanathan K., Shields S., Hipperson M., Reading F., Saunders J., Harper G., Honan W., Gill L., Stanley J., Vernon N., Skinner A., McCann P., Walker R., Edmonds P., O’Hanlon S., Wood B., Hand A., Robinson L., Liddle J., Bolam D., Raha S., Ebebezer L., Thompson S., Pall H., Praamstra P., Crouch R., Healy K., Johnson M., Jenkinson M., Abdel-Hafiz A., Al-Modaris F., Dutta S., Mallik T., Mondal B., Roberts J., Sinha S., Amar K., Atkins S., Devadason G., Martin A., Cox C., Malone T., Fenwick G., Gormley K., Gutowski N., Harris S., Harrower T., Hemsley A., James M., Jeffreys M.O., Pearce V., Sheridan R., Sword J., Zeman A., Soper C., Vassallo J., Bennett J., Lyell V., Robertson D., Howcroft D., Mugweni K., Stephens A., Whelan E., Wright A., Chamberlain J., Padiachy D., Marigold J., Lee J., Roberts H., Adams J., Dulay J., Evans S., Frankel J., Gove R., Turner G., Mallik N., McElwaine T., Morgan S., Phipps H., Pressly V., Queen V., Tan R., Grossett D., Macphee G., Vennard C., Rektorova I., Dhakam Z., Carey G., Castledon B., Sunderland C., Kalcantera E., Long C., Mandal B., Martin V., Nari R., Nicholas V., Moffitt V., Hammans S., Rice-Oxley M., Webb J., Franks S., Cooper S., Hussain M., Solanki T., Darch W., Homan J., Sharratt D., Griggs G., Kendall G., Ford A., Stocker K., Strens L., Grubneac A., Ponsford J., Teare L., Moore A.P., O’Brien I., Watling D., Wyatt L., Rizvi S., Walker E., Berry G., Russell N., Rashed K., Baker K., Qadiri M.R., Buckley C., Bulley S., Gibbons D., Goodland R., Heywood P., Jones L., Martin L., Rowland-Axe R., Stone A., Whittuck M.R. (2022). Long-term Effectiveness of Adjuvant Treatment With Catechol-O-Methyltransferase or Monoamine Oxidase B Inhibitors Compared With Dopamine Agonists Among Patients With Parkinson Disease Uncontrolled by Levodopa Therapy.. JAMA Neurol..

[19] van Kessel S.P., Frye A.K., El-Gendy A.O., Castejon M., Keshavarzian A., van Dijk G., El Aidy S. (2019). Gut bacterial tyrosine decarboxylases restrict levels of levodopa in the treatment of Parkinson’s disease.. Nat. Commun..

[20] Zhang K., Ni Y. (2014). Tyrosine decarboxylase from Lactobacillus brevis: Soluble expression and characterization.. Protein Expr. Purif..

[21] Perez M., Calles-Enríquez M., Nes I., Martin M.C., Fernandez M., Ladero V., Alvarez M.A. (2015). Tyramine biosynthesis is transcriptionally induced at low pH and improves the fitness of Enterococcus faecalis in acidic environments.. Appl. Microbiol. Biotechnol..

[22] Adibi S.A., Mercer D.W. (1973). Protein digestion in human intestine as reflected in luminal, mucosal, and plasma amino acid concentrations after meals.. J. Clin. Invest..

[23] Abrams W.B., Coutinho C.B., Leon A.S., Spiegel H.E. (1971). Absorption and metabolism of levodopa.. JAMA.

[24] Maini R.V., Bess E.N., Bisanz J.E., Turnbaugh P.J., Balskus E.P. (2019). Discovery and inhibition of an interspecies gut bacterial pathway for Levodopa metabolism.. Science.

[25] Whitfield A.C., Moore B.T., Daniels R.N. (2014). Classics in chemical neuroscience: levodopa.. ACS Chem. Neurosci..

[26] Bisanz J.E., Soto-Perez P., Lam K.N., Bess E.N., Haiser H.J., Allen-Vercoe E., Rekdal V.M., Balskus E.P., Turnbaugh P.J. (2018). Illuminating the microbiome’s dark matter: a functional genomic toolkit for the study of human gut Actinobacteria.. bioRxiv.

[27] Martínez-del Campo A., Bodea S., Hamer H.A., Marks J.A., Haiser H.J., Turnbaugh P.J., Balskus E.P. (2015). Characterization and detection of a widely distributed gene cluster that predicts anaerobic choline utilization by human gut bacteria.. MBio.

[28] van Kessel S.P., de Jong H.R., Winkel S.L., van Leeuwen S.S., Nelemans S.A., Permentier H., Keshavarzian A., El Aidy S. (2020). Gut bacterial deamination of residual levodopa medication for Parkinson’s disease.. BMC Biol..

[29] Donia M.S., Fischbach M.A. (2015). Small molecules from the human microbiota.. Science.

[30] Barker H.A. (1981). Amino acid degradation by anaerobic bacteria.. Annu. Rev. Biochem..

[31] Yvon M., Thirouin S., Rijnen L., Fromentier D., Gripon J.C. (1997). An aminotransferase from Lactococcus lactis initiates conversion of amino acids to cheese flavor compounds.. Appl. Environ. Microbiol..

[32] Nierop Groot M.N., de Bont J.A.M. (1998). Conversion of phenylalanine to benzaldehyde initiated by an aminotransferase in lactobacillus plantarum.. Appl. Environ. Microbiol..

[33] Elsden S.R., Hilton M.G., Waller J.M. (1976). The end products of the metabolism of aromatic amino acids by clostridia.. Arch. Microbiol..

[34] Dodd D., Spitzer M.H., Van Treuren W., Merrill B.D., Hryckowian A.J., Higginbottom S.K., Le A., Cowan T.M., Nolan G.P., Fischbach M.A., Sonnenburg J.L. (2017). A gut bacterial pathway metabolizes aromatic amino acids into nine circulating metabolites.. Nature.

[35] Bansal T., Alaniz R.C., Wood T.K., Jayaraman A. (2010). The bacterial signal indole increases epithelial-cell tight-junction resistance and attenuates indicators of inflammation.. Proc. Natl. Acad. Sci. USA.

[36] Schiering C., Wincent E., Metidji A., Iseppon A., Li Y., Potocnik A.J., Omenetti S., Henderson C.J., Wolf C.R., Nebert D.W., Stockinger B. (2017). Feedback control of AHR signalling regulates intestinal immunity.. Nature.

[37] Bhattarai Y., Williams B.B., Battaglioli E.J., Whitaker W.R., Till L., Grover M., Linden D.R., Akiba Y., Kandimalla K.K., Zachos N.C., Kaunitz J.D., Sonnenburg J.L., Fischbach M.A., Farrugia G., Kashyap P.C. (2018). Gut Microbiota-Produced Tryptamine Activates an Epithelial G-Protein-Coupled Receptor to Increase Colonic Secretion.. Cell Host Microbe.

[38] Venkatesh M., Mukherjee S., Wang H., Li H., Sun K., Benechet A.P., Qiu Z., Maher L., Redinbo M.R., Phillips R.S., Fleet J.C., Kortagere S., Mukherjee P., Fasano A., Le Ven J., Nicholson J.K., Dumas M.E., Khanna K.M., Mani S. (2014). Symbiotic bacterial metabolites regulate gastrointestinal barrier function via the xenobiotic sensor PXR and Toll-like receptor 4.. Immunity.

[39] Morgan J.P., Bianchine J.R., Spiegel H.E., Rivera-Calimlim L., Hersey R.M. (1971). Metabolism of levodopa in patients with Parkinson’s disease. Radioactive and fluorometric assays.. Arch. Neurol..

[40] Bianchine J.R., Messiha F.S., Hsu T.H. (1972). Peripheral aromatic L-amino acids decarboxylase inhibitor in parkinsonism. II. Effect on metabolism of L-2-14C-dopa.. Clin. Pharmacol. Ther..

[41] Sasahara K., Nitanai T., Habara T., Kojima T., Kawahara Y., Morioka T., Nakajima E. (1981). Dosage form design for improvement of bioavailability of levodopa IV: Possible causes of low bioavailability of oral levodopa in dogs.. J. Pharm. Sci..

[42] Goldin B.R., Peppercorn M.A., Goldman P. (1973). Contributions of host and intestinal microflora in the metabolism of L-dopa by the rat.. J. Pharmacol. Exp. Ther..

[43] Dickert S., Pierik A.J., Linder D., Buckel W. (2000). The involvement of coenzyme A esters in the dehydration of (R)-phenyllactate to (E)-cinnamate by Clostridium sporogenes.. Eur. J. Biochem..

[44] Dickert S., Pierik A.J., Buckel W. (2002). Molecular characterization of phenyllactate dehydratase and its initiator from Clostridium sporogenes.. Mol. Microbiol..

[45] Roager H.M., Hansen L.B.S., Bahl M.I., Frandsen H.L., Carvalho V., Gøbel R.J., Dalgaard M.D., Plichta D.R., Sparholt M.H., Vestergaard H., Hansen T., Sicheritz-Pontén T., Nielsen H.B., Pedersen O., Lauritzen L., Kristensen M., Gupta R., Licht T.R. (2016). Colonic transit time is related to bacterial metabolism and mucosal turnover in the gut.. Nat. Microbiol..

[46] Jin J.S., Hattori M. (2012). Isolation and characterization of a human intestinal bacterium Eggerthella sp. CAT-1 capable of cleaving the C-ring of (+)-catechin and (-)-epicatechin, followed by p-dehydroxylation of the B-ring.. Biol. Pharm. Bull..

[47] Haiser H.J., Gootenberg D.B., Chatman K., Sirasani G., Balskus E.P., Turnbaugh P.J. (2013). Predicting and manipulating cardiac drug inactivation by the human gut bacterium Eggerthella lenta.. Science.

[48] Fasano A., Visanji N.P., Liu L.W.C., Lang A.E., Pfeiffer R.F. (2015). Gastrointestinal dysfunction in Parkinson’s disease.. Lancet Neurol..

[49] Panagamuwa B., Kumar D., Ortiz J., Keighley M.R.B. (2005). Motor abnormalities in the terminal ileum of patients with chronic idiopathic constipation.. Br. J. Surg..

[50] Van Der Sijp J.R.M., Kamm M.A., Nightingale J.M.D., Britton K.E., Granowska M., Mather S.J., Akkermans L.M.A., Lennard-Jones J.E. (1993). Disturbed gastric and small bowel transit in severe idiopathic constipation.. Dig. Dis. Sci..

[51] Broadley K.J. (2010). The vascular effects of trace amines and amphetamines.. Pharmacol. Ther..

[52] Lindemann L., Hoener M.C. (2005). A renaissance in trace amines inspired by a novel GPCR family.. Trends Pharmacol. Sci..

[53] Nagatsu T., Nakashima A., Ichinose H., Kobayashi K. (2019). Human tyrosine hydroxylase in Parkinson’s disease and in related disorders.. J. Neural Transm. (Vienna).

[54] Hayashi H., Mizuguchi H., Kagamiyama H. (1993). Rat liver aromatic L-amino acid decarboxylase: Spectroscopic and kinetic analysis of the coenzyme and reaction intermediates.. Biochemistry.

[55] Kim, Cho K.H., Shin M.S., Lee J.M., Cho H.S., Kim C.J., Shin D.H., Yang H.J. (2014). Berberine prevents nigrostriatal dopaminergic neuronal loss and suppresses hippocampal apoptosis in mice with Parkinson’s disease.. Int. J. Mol. Med..

[56] Kwon I.H., Choi H.S., Shin K.S., Lee B.K., Lee C.K., Hwang B.Y., Lim S.C., Lee M.K. (2010). Effects of berberine on 6-hydroxydopamine-induced neurotoxicity in PC12 cells and a rat model of Parkinson’s disease.. Neurosci. Lett..

[57] Wang Y., Tong Q., Ma S.R., Zhao Z.X., Pan L.B., Cong L., Han P., Peng R., Yu H., Lin Y., Gao T.L., Shou J.W., Li X.Y., Zhang X.F., Zhang Z.W., Fu J., Wen B.Y., Yu J.B., Cao X., Jiang J.D. (2021). Oral berberine improves brain dopa/dopamine levels to ameliorate Parkinson’s disease by regulating gut microbiota.. Signal Transduct. Target. Ther..

[58] Feng R., Shou J.W., Zhao Z.X., He C.Y., Ma C., Huang M., Fu J., Tan X.S., Li X.Y., Wen B.Y., Chen X., Yang X.Y., Ren G., Lin Y., Chen Y., You X.F., Wang Y., Jiang J.D. (2015). Transforming berberine into its intestine-absorbable form by the gut microbiota.. Sci. Rep..

[59] Kim H.L., Park Y.S. (2010). Maintenance of cellular tetrahydrobiopterin homeostasis.. BMB Rep..

[60] Surwase S.N., Jadhav J.P. (2011). Bioconversion of l-tyrosine to l-DOPA by a novel bacterium Bacillus sp. JPJ.. Amino Acids.

[61] Houck D.R., Hanners J.L., Unkefer C.J., van Kleef M.A.G., Duine J.A. (1989). PQQ: Biosynthetic studies inMethylobacterium AM1 andHyphomicrobium X using specific13C labeling and NMR.. Antonie van Leeuwenhoek.

[62] Muñoz A.J., Hernández-Chávez G., de Anda R., Martínez A., Bolívar F., Gosset G. (2011). Metabolic engineering of Escherichia coli for improving l-3,4-dihydroxyphenylalanine (l-DOPA) synthesis from glucose.. J. Ind. Microbiol. Biotechnol..

[63] Connolly B.S., Lang A.E. (2014). Pharmacological treatment of Parkinson disease: a review.. JAMA.

[64] Gey K.F., Pletscher A. (1964). Distribution and metabolism of DL -3,4-dihydroxy[2-14C]-phenylalanine in rat tissues.. Biochem. J..

[65] Bergmann S., Curzon G., Friedel J., Godwin-Austen R.B., Marsden C.D., Parkes J.D. (1974). The absorption and metabolism of a standard oral dose of levodopa in patients with Parkinsonism.. Br. J. Clin. Pharmacol..

[66] Cotzias G.C., Papavasiliou P.S., Ginos J., Steck A., Düby S. (1971). Metabolic modification of Parkinson’s disease and of chronic manganese poisoning.. Annu. Rev. Med..

[67] Goldenberg M.M. (2008). Medical management of Parkinson’s disease.. P&T.

[68] Burkhard P., Dominici P., Borri-Voltattorni C., Jansonius J.N., Malashkevich V.N. (2001). Structural insight into Parkinson’s disease treatment from drug-inhibited DOPA decarboxylase.. Nat. Struct. Biol..

[69] Montioli R., Voltattorni C.B., Bertoldi M. (2016). Parkinson’s Disease: Recent Updates in the Identification of Human Dopa Decarboxylase Inhibitors.. Curr. Drug Metab..

[70] Parkinson Disease Agents (2012). LiverTox: Clinical and Research Information on Drug-Induced Liver Injury..

[71] Fabbri M., Ferreira J.J., Rascol O. (2022). COMT Inhibitors in the Management of Parkinson’s Disease.. CNS Drugs.

[72] Alonso C.A., Luquin P.R., García, Ruiz-Espiga P., Burguera J.A., Campos Arillo V., Castro A., Linazasoro G., López Del Val J., Vela L., Martínez Castrillo J.C. (2014). Dopaminergic agonists in Parkinson’s disease.. Neurologia.

[73] Latt M.D., Lewis S., Zekry O., Fung V.S.C. (2019). Factors to consider in the selection of dopamine agonists for older persons with Parkinson’s disease.. Drugs Aging.

[74] Tan Y.Y., Jenner P., Chen S.D. (2022). Monoamine oxidase-B inhibitors for the treatment of Parkinson’s disease: Past, present, and future.. J. Parkinsons Dis..

[75] Nafisah W., Najman A.H., Hamizah R., Azmin S., Rabani R., Shah S., Norlinah M. (2013). High prevalence of Helicobacter pylori infection in Malaysian Parkinson’s disease patients.. Research and Reviews in Parkinsonism.

[76] Pierantozzi M., Pietroiusti A., Galante A., Sancesario G., Lunardi G., Fedele E., Giacomini P., Stanzione P. (2001). Helicobacter pylori-induced reduction of acute levodopa absorption in parkinson’s disease patients.. Ann. Neurol..

[77] Rees K., Stowe R., Patel S., Ives N., Breen K., Clarke C.E., Ben-Shlomo Y. (2011). Helicobacter pylori eradication for Parkinson’s disease.. Cochrane Libr..

[78] Pierantozzi M., Pietroiusti A., Brusa L., Galati S., Stefani A., Lunardi G., Fedele E., Sancesario G., Bernardi G., Bergamaschi A., Magrini A., Stanzione P., Galante A. (2006). Helicobacter pylori eradication and l-dopa absorption in patients with PD and motor fluctuations.. Neurology.

[79] Bjarnason I.T., Charlett A., Dobbs R.J., Dobbs S.M., Ibrahim M.A.A., Kerwin R.W., Mahler R.F., Oxlade N.L., Peterson D.W., Plant J.M., Price A.B., Weller C. (2005). Role of chronic infection and inflammation in the gastrointestinal tract in the etiology and pathogenesis of idiopathic parkinsonism. Part 2: response of facets of clinical idiopathic parkinsonism to Helicobacter pylori eradication. A randomized, double-blind, placebo-controlled efficacy study.. Helicobacter.

[80] Beales I.L.P., Calam J. (1998). Interleukin 1β and tumour necrosis factor α inhibit acid secretion in cultured rabbit parietal cells by multiple pathways.. Gut.

[81] El-Omar E.M. (2001). The importance of interleukin 1β in Helicobacter pylori associated disease.. Gut.

[82] Takashima M., Furuta T., Hanai H., Sugimura H., Kaneko E. (2001). Effects of Helicobacter pylori infection on gastric acid secretion and serum gastrin levels in Mongolian gerbils.. Gut.

[83] Feldman M., Cryer B., Lee E. (1998). Effects of Helicobacter pylori gastritis on gastric secretion in healthy human beings.. Am. J. Physiol. Gastrointest. Liver Physiol..

[84] Thor P., Lorens K., Tabor S., Herman R., Konturek J.W., Konturek S.J. (1996). Dysfunction in gastric myoelectric and motor activity in Helicobacter pylori positive gastritis patients with non-ulcer dyspesia.. J. Physiol. Pharmacol..

[85] Miyaji H., Azuma T., Ito S., Abe Y., Ono H., Suto H., Ito Y., Yamazaki Y., Kohli Y., Kuriyama M. (1999). The effect of Helicobacter pylori eradication therapy on gastric antral myoelectrical activity and gastric emptying in patients with non-ulcer dyspepsia.. Aliment. Pharmacol. Ther..

[86] Doherty N.C., Tobias A., Watson S., Atherton J.C. (2009). The effect of the human gut-signalling hormone, norepinephrine, on the growth of the gastric pathogen Helicobacter pylori.. Helicobacter.

[87] Yang J.C., Lu C.W., Lin C.J. (2014). Treatment of Helicobacter pylori infection: Current status and future concepts.. World J. Gastroenterol..

[88] Gasbarrini A., Lauritano E.C., Gabrielli M., Scarpellini E., Lupascu A., Ojetti V., Gasbarrini G. (2007). Small intestinal bacterial overgrowth: diagnosis and treatment.. Dig. Dis..

[89] Gabrielli M., Bonazzi P., Scarpellini E., Bendia E., Lauritano E.C., Fasano A., Ceravolo M.G., Capecci M., Rita Bentivoglio A., Provinciali L., Tonali P.A., Gasbarrini A. (2011). Prevalence of small intestinal bacterial overgrowth in Parkinson’s disease.. Mov. Disord..

[90] Wanitschke R., Ammon H.V. (1978). Effects of dihydroxy bile acids and hydroxy fatty acids on the absorption of oleic acid in the human jejunum.. J. Clin. Invest..

[91] Nucera G., Gabrielli M., Lupascu A., Lauritano E.C., Santoliquido A., Cremonini F., Cammarota G., Tondi P., Pola P., Gasbarrini G., Gasbarrini A. (2005). Abnormal breath tests to lactose, fructose and sorbitol in irritable bowel syndrome may be explained by small intestinal bacterial overgrowth.. Aliment. Pharmacol. Ther..

[92] Spencer R.P. (1969). Intestinal absorption of amino acids. Current concepts.. Am. J. Clin. Nutr..

[93] Dobbs R.J., Charlett A., Dobbs S.M., Weller C., Peterson D.W. (2000). Parkinsonism: differential age-trend in Helicobacter pylori antibody.. Aliment. Pharmacol. Ther..

[94] Zimmermann M., Zimmermann-Kogadeeva M., Wegmann R., Goodman A.L. (2019). Mapping human microbiome drug metabolism by gut bacteria and their genes.. Nature.

[95] Gatto M., Fernandez Pardal M., Melero M., Zurru C., Scorticati C., Micheli F. (1994). L-dopa malabsorption in a parkinsonian patient with Strongyloides stercoralis duodenitis.. Clin. Neuropharmacol..

[96] Pereira C.I., Matos D., San Romão M.V., Barreto Crespo M.T. (2009). Dual role for the tyrosine decarboxylation pathway in Enterococcus faecium E17: response to an acid challenge and generation of a proton motive force.. Appl. Environ. Microbiol..

[97] Fallingborg J. (1999). Intraluminal pH of the human gastrointestinal tract.. Dan. Med. Bull..

